# Bittersweet: how hyperglycemia exacerbates vitiligo progression through the succinate/SUCNR1 axis

**DOI:** 10.1172/JCI207416

**Published:** 2026-06-15

**Authors:** Kaitlyn G. O’Donnell, I. Caroline Le Poole

**Affiliations:** 1 Indiana University School of Medicine, Indianapolis, Indiana, USA.; 2 Departments of Dermatology and Microbiology–Immunology, Feinberg School of Medicine, Northwestern University, Chicago, Illinois, USA.

## Abstract

Vitiligo is a depigmenting disease marked by progressive T cell–driven destruction of melanocytes in the skin, hair, and mucosa. While vitiligo is known to be a T cell–mediated autoimmune disease, its triggers have remained poorly understood and treatment options limited. In this issue of the *JCI*, Kang et al. demonstrated how hyperglycemia exacerbates vitiligo progression through the succinate/SUCNR1 axis. These findings identify succinate as a potential biomarker for disease activity and highlight an independent pathway for targeting in therapeutic intervention. More broadly, the findings linking succinate and glucose metabolism to vitiligo suggest that lifestyle factors could be modified to slow development of vitiligo and other autoimmune diseases linked to succinate.

## Vitiligo: metabolic factors play a role

Vitiligo is an autoimmune disease characterized by T cell–mediated destruction of melanocytes, leading to the progressive loss of pigment in the skin, hair, and mucosa (1). While elevated T cell activation is well documented in vitiligo and is one of only few established biomarkers for disease activity, the underlying cause of this activation remains unclear. Existing therapies for vitiligo may slow disease progression but are often unable to restore pigment that has been lost. Emerging evidence continues to uncover the pathophysiology of vitiligo as an interplay of genetic, autoimmune, and environmental factors. There is increasing appreciation for the impact of metabolic influences in vitiligo pathology, including evidence for disruptions in glucose metabolism (2).

The study presented by Kang et al. (3) in this issue of the *JCI* describes a role for hyperglycemia in disease development. This work offers a glimpse into the intriguing opportunity to make lifestyle adjustments that might alleviate disease progression in patients.

## A new biomarker of disease activity

First, the authors substantiated observations that patients with vitiligo are at increased risk of developing hyperglycemia (4, 5). In a hospital-based study, Kang et al. observed that not only were participants with vitiligo at increased risk of hyperglycemia, but hyperglycemia also correlated with disease activity and severity in the subset of participants with vitiligo. The authors found several TCA cycle intermediates to be elevated in blood and, importantly, in the skin of patients with vitiligo. Among these intermediates, succinate was identified as an apparent driver of disease. This finding is an advancement in itself, as it suggests that circulating succinate may be a valuable biomarker of disease activity and may be of use in future clinical trials. Two previously established biomarkers for vitiligo, increased IFN-γ in blister rooftops (6) and T cell infiltration in advancing skin lesions (7), require invasive methods of detection, and the relatively simple measurement of serum succinate could be an advantage in many clinical trial settings.

## Succinate in the driver’s seat

Next, the authors used a mouse model of vitiligo to unveil a potential role for the Krebs cycle intermediate in vitiligo disease development. To do this, Kang et al. induced vitiligo in the presence of hyperglycemia in mice lacking the receptor for succinate, SUCNR1, and observed that loss of succinate/SUCNR1 signaling attenuated vitiligo development.

## T cells slow down without a succinate signal

Leaning on the known role of T cells in disease development, the authors next measured T cell activation in SUCNR1-deficient mice and found that the T cells were significantly subdued, as measured by reduced activation marker expression. Again, this is an important observation, as an underlying cause for elevated T cell activation in vitiligo has remained quite elusive to date. It has been reported that succinate acts on macrophages and dendritic cells while receptor expression is minimal on lymphocytes, so succinate impact on T cell activation is likely indirect (8).

## SUCNR1 makes keratinocytes attractive

**Figure 1:**
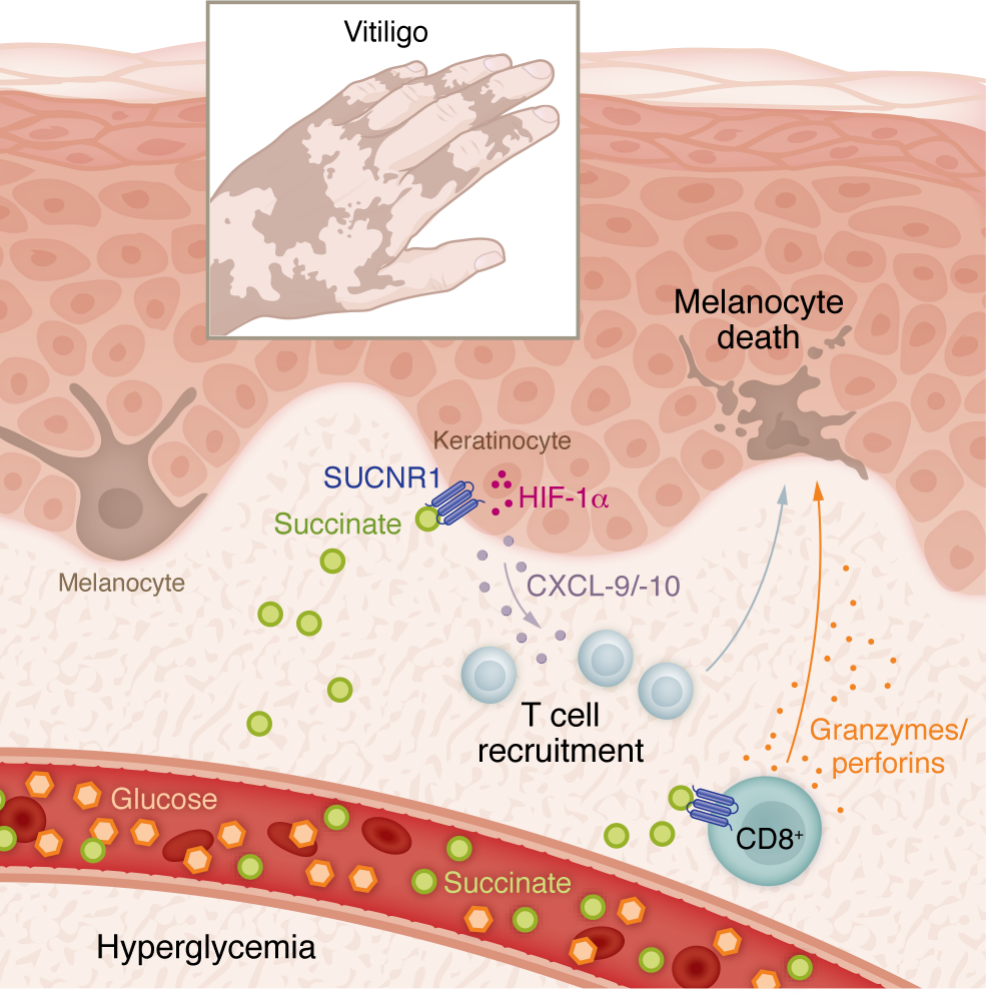
Hyperglycemia exacerbates vitiligo by way of the succinate/SUCNR1 axis. Kang et al. reported that hyperglycemia is associated with succinate accumulation in the serum and skin (3). They further showed that succinate interaction with SUCNR1 on both T cells and keratinocytes contributes to disease. CD8^+^ T cells in patients with hyperglycemia-complicated vitiligo expressed SUCNR1 at higher rates and demonstrated increased activation and effector function in response to succinate, leading to escalated melanocyte cell death. Increased keratinocyte production of CXCL9 and -10 via stabilization of hypoxia-inducible factor-1α (HIF-1α) promoted further recruitment of CD8^+^ T cells to the skin.

Keratinocytes that surround and interact with pigment-producing melanocytes also contribute to vitiligo pathophysiology. In analyses of previously published human datasets, Kang et al. observed elevated succinate in vitiligo lesions as well as increased expression of SUCNR1 in keratinocytes. Keratinocytes were also affected by the loss of SUCNR1 in the mouse models engaged in their study. The authors went to great lengths to reveal that exposure to succinate drives stabilized HIF-1α expression and translocation to the nucleus. Nuclear HIF-1α, in turn, drives the transcription, production, and secretion of chemotactic factors CXCL9 and -10 from epidermal keratinocytes. These two chemokines have already been solidly established as recruitment factors for additional T cells to the skin (9, 10). Thus, the succinate-driven increase in CXCL9 and -10 causes a substantial increase in melanocyte-reactive T cells in the skin that can further accelerate and aggravate vitiligo development (Figure 1). Taken together, Kang et al. have unveiled an intriguing pathway linking hyperglycemia to progressive depigmentation in vitiligo.

## A potential link to diabetes

Metabolic induction of hyperglycemia observed in this study is reminiscent of conditions in type 2 diabetes, which can develop in response to a high-fat diet. However, the expected weight gain associated with such diet is not observed in patients with vitiligo (11). In fact, prior studies suggest that patients with active disease select a diet with reduced fat intake, a dietary shift associated with specific changes to the gut microbiome (12). This might suggest that the hyperglycemia observed in vitiligo is not so much related to type 2 as to type 1 diabetes, which is itself driven by autoimmune pathology, though such speculation extends beyond the reach of the work presented here (13).

## Succinate in other settings

The reach of Kang et al.’s findings may stretch beyond vitiligo, as different autoimmune conditions have been liked to succinate metabolism. Excess succinate has been implicated in the pathogenesis of rheumatoid arthritis, as its accumulation in the joint synovial fluid elicits IL-1β secretion from SUCNR1-expressing macrophages, thereby exacerbating inflammation (14). Moreover, in inflammatory bowel disease, elevated serum and intestinal succinate levels correlate with increased SUCNR1 expression, leading to receptor-modulated expression of pro-inflammatory cytokines IL-1β, IL-16, and TNF-α as well as increased expression of TGF-β, leading to fibrosis (15).

## Inspiration for new vitiligo treatments

Currently available therapies for vitiligo (16) include topical steroids, topical calcineurin inhibitors, narrow-band UVB therapy, and, most recently, topical JAK inhibitors (17); however, much potential remains for an improvement in available therapeutics, as repigmentation rates are variable (16). Kang et al.’s study provides a rationale for exploring succinate receptor blockade to reduce keratinocyte recruitment and activation of melanocyte-reactive T cells in the development of treatments for vitiligo (3). Indeed, targeting the succinate/SUCNR1 axis would offer a fresh, alternative strategy for approaching vitiligo treatment. Conversely, existing therapies for diabetes mellitus, such as PPAR-γ agonists, are currently being investigated as options for potential vitiligo treatments and could offer a more favorable side effect profile compared with immunosuppressive agents (2). Regardless of the specific strategy under investigation, this metabolism-focused approach could allow for the development of multiple therapeutic agents with hopes of augmenting treatment and minimizing disease. Kang et al.’s findings also come at an opportune time considering the increasing worldwide prevalence of diabetes, with an estimated 11% of the adult population affected worldwide currently and a projected 13% of patients likely to be affected by 2050 (18). This growing percentage is not negligible in the setting of hyperglycemia-complicated vitiligo, and proper surveillance and enforcement of glycemic control in vitiligo could greatly benefit disease outcomes.

## A call for understanding lifestyle impact

The study further places a spotlight on modifiable lifestyle factors that can exacerbate vitiligo. While it is known that a well-balanced diet is essential to patient health, we are especially reminded of its importance in the context of vitiligo and other autoimmune diseases. Dietary interventions directed at the gut microbiome, particularly those favoring succinate-consuming microbes, may support immune function in the gut and beyond (19). With succinate-producing microbes promoting inflammation, the combination of observations provides an opportunity to investigate how the microbiome, succinate metabolism, and autoimmunity are intertwined.

## What about Tregs?

A potential limitation to the study involves the use of the melanoma-Treg mouse model. This model relies on depletion of Tregs to induce vitiligo development in mice so that cytotoxic T cells, primed by an inoculation of melanoma cells, can function uninhibited. Whether Tregs contribute succinate/SUCNR1-mediated effects in the context of vitiligo remains unknown. Indeed, under circumstances found in the tumor microenvironment, excess succinate has been shown to stimulate Tregs (20). In a Treg-sufficient model of vitiligo, these Tregs may also be impacted by succinate and counter some of the detrimental outcomes described above. This limitation is mitigated at least in part by reduced cutaneous Treg activity reported in vitiligo as well (21).

## It’s bittersweet

Taken together, the study by Kang et al. highlights the role of succinate, a TCA intermediate and immune-modulating agent in vitiligo, and provides exciting leads for measures of disease activity and its future interventions.

## Conflict of interest

ICLP is the chief scientific officer for Temprian Therapeutics Inc and the chief innovations officer of Temprian Oncology Inc., two Northwestern spinoff companies launched to enable HSP70i_Q435A_ treatment for immune conditions including vitiligo and monobenzone-derivative nanoparticles for the treatment of melanoma, respectively.

## Funding support

- ICLP by Leo Foundation grant LF-OC-23-001288.

## Version 1 06/15/2026

Electronic publication
